# Impact of Particle Size on the Nonlinear Magnetic Response of Iron Oxide Nanoparticles during Frequency Mixing Magnetic Detection

**DOI:** 10.3390/s24134223

**Published:** 2024-06-29

**Authors:** Ali Mohammad Pourshahidi, Neha Jean, Corinna Kaulen, Simon Jakobi, Hans-Joachim Krause

**Affiliations:** 1 Institute of Biological Information Processing, Bioelectronics (IBI-3), Forschungszentrum Jülich, 52425 Jülich, Germany; n.jean@fz-juelich.de; 2 Ostbayerische Technische Hochschule Regensburg, 93053 Regensburg, Germany; corinna.kaulen@oth-regensburg.de; 3 RWTH Aachen, Institute of Inorganic Chemistry, 52074 Aachen, Germany

**Keywords:** frequency mixing magnetic detection, magnetic nanoparticles, magnetic particle spectroscopy, magnetic biosensing, thermal decomposition, iron oxide nanoparticles, MNP characterization

## Abstract

Magnetic nanoparticles (MNPs), particularly iron oxide nanoparticles (IONPs), play a pivotal role in biomedical applications ranging from magnetic resonance imaging (MRI) enhancement and cancer hyperthermia treatments to biosensing. This study focuses on the synthesis, characterization, and application of IONPs with two different size distributions for frequency mixing magnetic detection (FMMD), a technique that leverages the nonlinear magnetization properties of MNPs for sensitive biosensing. IONPs are synthesized through thermal decomposition and subsequent growth steps. Our findings highlight the critical influence of IONP size on the FMMD signal, demonstrating that larger particles contribute dominantly to the FMMD signal. This research advances our understanding of IONP behavior, underscoring the importance of size in their application in advanced diagnostic tools.

## 1. Introduction

In the evolving landscape of biomedical research, magnetic nanoparticles (MNPs) have emerged as a cornerstone for their significant contributions across various domains [1,2]. These include enhanced contrast in magnetic resonance imaging (MRI) [3,4], tracers in magnetic particle imaging (MPI) [5,6,7], hyperthermia treatment for cancer [8,9,10,11], and biosensing [12,13,14]. In MRI, they improve the contrast between different tissue types, enabling an enhanced visualization of anatomical structures and pathological conditions [15,16]. In magnetic hyperthermia, these nanoparticles can be heated by an external magnetic field to ablate cancerous cells, offering a minimally invasive treatment option that can be focused on the tumor site, thus sparing surrounding healthy tissues [17].

**Figure 1:**
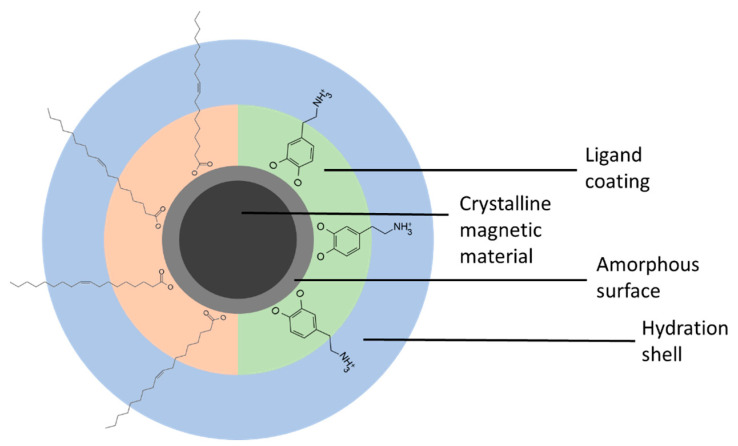
Schematic drawing of a single magnetic nanoparticle, showing the crystalline core, which is surrounded by an amorphous shell, the ligand coating, and the hydration shell. From the ligands here, oleic acid, as an example of a hydrophobic ligand, and dopamine, as a hydrophilic coating, is shown. Not drawn to scale.

The most famous and, due to their good bioavailability and low toxicity, the most utilized class of MNPs are iron oxide nanoparticles (IONPs). As illustrated in Figure 1, they contain a crystalline core that is the main contributor to the magnetic properties of the particle, an amorphous surface layer that is less magnetically active, a ligand coating, and, in liquid solution, a hydration shell [18,19]. This model serves as a fundamental understanding of the IONP structure, emphasizing the importance of each component in influencing the overall magnetic behavior. Further surface functionalization through the conjugation of specific biomolecules on the surface of the particles consequently enables their use in biomedical research fields.

In magnetic biosensing, IONPs have shown profound potential for the detection and quantification of biomolecules, thus providing a powerful tool for the early diagnosis and monitoring of diseases [20,21,22,23,24]. Depending on the applied method, the IONPs have to fulfill specific requirements regarding their size to achieve an optimal output signal. For contrast enhancement of the transverse relaxation (*T*_2_) signal in MRI, the contrast increases with the increasing IONP size [25]. Moreover, in magnetic particle spectroscopy (MPS), the size of the analyzed IONPs contributes significantly to the obtained signal [26,27]. This emphasizes the imperative for stringent quality control throughout the synthesis and characterization processes of MNPs, ensuring their efficacy for intended applications.

A notable advancement in biosensing technology is the application of frequency mixing magnetic detection (FMMD), which has shown promising capabilities in point-of-care monitoring [28,29,30]. FMMD offers unique advantages, for example, permitting the concurrent identification of various analytes such as deoxyribonucleic acid (DNA) [31], toxins, and viruses [32,33]. The technology provides the ability for the simultaneous measurement of two or more analytes, the so-called multiplex detection [34,35,36,37], and even permits an evaluation of the core size distribution of the magnetic nanoparticles [26,38]. These features make FMMD an invaluable tool in refining biosensing methodologies, especially in environments where quick and accurate diagnostics are crucial.

FMMD is a technique that exploits the nonlinear magnetization properties of MNPs under the influence of dual-frequency magnetic excitation. This method involves the application of two alternating magnetic fields at different frequencies, typically a low-frequency driving field and a high-frequency probing field. When these fields are applied to a sample containing MNPs, the particles’ nonlinear magnetic response generates mixing harmonics at the sum and difference of the original frequencies [29]. The detection and analysis of these harmonics provide a sensitive measure of the MNPs’ characteristics with respect to the variation in parameters, such as the core size distribution, hydrodynamic size, and immobilization state. The FMMD technique is particularly advantageous for the selective quantification and characterization of MNPs due to its ability to discern the unique magnetic signatures of different particle types, provided that they exhibit different core sizes [34]. The presence of a static offset magnetic field further improves the detection capabilities, allowing for the identification of even and odd harmonics and improving the diagnostic capabilities of the technique [39]. The precision and versatility of this method make it a valuable tool in the field of biosensing, where a detailed understanding of nanoparticle behavior is crucial for the development of advanced diagnostic tests.

For biosensing applications, a suitable response signal is needed that can contribute to both the detection sensitivity and also the dynamic detection range. Different contributing aspects to the FMMD signal have been evaluated in [40] from a dynamic simulation approach. The results show that the core size strongly affects the FMMD signal. Moreover, the simulation of the core size dependency of the signals suggests that when assuming a very narrow distribution width *σ* of 0.05, the particles below *d*_0_ = 16 nm generate almost no signal, and it is recommended that larger particles are used. MNPs suitable for nonlinear magnetic detection have to exhibit specific properties, like high crystallinity, high monodispersity, and overall superparamagnetism. For applications in biomedicine, the particles should also be non-toxic. For medical applications, non-toxic IONPs, consisting of magnetite (Fe_3_O_4_) or maghemite (Fe_2_O_3_) are suitable. These materials can be synthesized by different methods, each offering a specific benefit for the characteristics of the obtained particles. The easiest method is the so-called coprecipitation method, first reported by Massart in 1981 [41]. Here, a solution of ferric and ferrous salts is mixed with a basic solution, leading to the precipitation of iron hydroxides, which, in the course of the reaction age, form magnetite nanoparticles. Although this method is fast and cheap, the obtained particles are polydisperse, show low crystallinity, and tend to form aggregates, which makes them not well suited for applications in FMMD. Another method for the synthesis of magnetite MNP is the thermal decomposition of iron-containing precursor salts at high temperatures of around 300 °C in organic solvent [42]. This method yields highly monodisperse IONPs coated with oleic acid, which renders the particles soluble only in organic solvents, e.g., hexane or toluene. IONPs obtained by thermal decomposition are highly crystalline, but 4 nm to 8 nm are also rather small. For applications where larger IONPs are required, a subsequent seed-mediated growth step can be performed to obtain IONPs with an increased size. The initially obtained small particles are employed in a second reaction step where the formed iron oxide material attaches preferentially to the seed particles, leading to larger-sized IONPs.

Here, we report on the application of differently sized magnetite IONPs synthesized by thermal decomposition for magnetic detection by FMMD. We analyzed their nonlinear magnetic response and compared their performance. By employing standard characterization methodologies, we showed experimentally how the particle size variation influences the FMMD signal and how precise the core size evaluation based on the FMMD technique is compared to other methods, i.e., dynamic light scattering (DLS) and transmission electron microscopy (TEM). This study allows us to experimentally evaluate the impact of the size distribution of the MNPs on the FMMD signal, specifically for the smaller size ranges. Our investigation not only enhances our comprehension of magnetic nanoparticle behavior but also propels forward the development of more sophisticated diagnostic tools for point-of-care applications.

## 2. Materials and Methods

### 2.1. Synthesis of Magnetic Nanoparticles

Benzylether (98%), 1,2-hexadecanediol (90%), and oleylamine were purchased from Sigma Aldrich; Fe(acac)_3_ (97%) was purchased from Merck KGaA; oleic acid (90%), hexane, ethanol, and toluene were received from Carl Roth GmbH + Co. KG; Karlsruhe, Germany. All chemicals were used without further purification. The thermal decomposition reactions were performed under a nitrogen atmosphere, and further purification of the IONPs was performed under ambient air.

#### 2.1.1. Synthesis of the Seed IONPs

The seed IONPs were synthesized by the thermal decomposition method and subsequent growth of the seed particles [43]. Briefly, a mixture of 2 mmol Fe(acac)_3_, 10 mmol 1,2-hexadecanediol, 6 mmol oleic acid, and 6 mmol oleylamine was dissolved in 20 mL of benzylether and heated under magnetic stirring to 200 °C with a heating rate of 4 °C/min in a three-neck round-bottom flask. The reaction mixture was kept at 200 °C for 2 h; during this time, the color of the mixture changed from dark red to deep brown. Then, the mixture was heated to reflux (298 °C) for one hour. After this time, the mixture was allowed to cool down to room temperature, and the formed IONPs were precipitated by the addition of 40 mL of ethanol. The precipitates were collected with a magnet, and the supernatant was discarded. The residues were washed two times with ethanol and then dissolved in hexane.

#### 2.1.2. Growth of the Seed IONPs

In total, 20 mg of the seed IONPs, 2 mmol of Fe(acac)_3_, 10 mmol of 1,2-hexadecanediol, 6 mmol of oleic acid, and 6 mmol of oleylamine, were dissolved in 20 mL of benzylether and heated to 200 °C for 2 h. Then, the mixture was heated to reflux (298 °C) and kept at this temperature for one hour. After cooling to room temperature, the IONPs were isolated by precipitation via the addition of 40 mL ethanol, which was washed several times with ethanol and redispersed in hexane.

#### 2.1.3. Fractionated Precipitation of IONPs

To receive monodisperse size fractions, the crude IONPs were submitted to fractionated precipitation. For this purpose, a small amount of ethanol was added (100 µL to 1 mL of solution) to the hexane solution of the IONPs. Ethanol represents a non-solvent and precipitates the largest fraction of the IONPs, while the smaller IONPs stay in the solution. The precipitated IONPs are sedimented by centrifugation (10,000× *g*/15 min). The supernatant is removed, and another precipitation step is performed by adding ethanol to the supernatant. After the addition of 300 µL of ethanol, all particles were precipitated, and the individual fractions were dissolved in hexane and characterized. Size fractionated samples with a uniform size and narrow size distribution (PDI < 0.1) were used for the subsequent growth steps.

### 2.2. Characterization of the Synthesized IONP

The size-fractionated IONPs were dissolved in hexane, and the obtained solution was used for DLS measurements. TEM samples were prepared by the deposition of 10 µL of this solution on a carbon-coated copper grid. The size and shape of the IONPs were characterized by transmission electron microscopy (TEM) using a Libra 200 Field Emission TEM (FE-TEM, Carl Zeiss AG, Oberkochen, Germany) operated at 200 keV. The hydrodynamic radius and the polydispersity index were characterized using LitesizerTM 500 and Kalliope Anton Paar software from Anton Paar, Germany. The iron content was measured by employing inductively coupled plasma optical emission spectroscopy (ICP-OES), iCAP7600 (Thermo Scientific™, Langerwehe, Germany).

### 2.3. Frequency Mixing Magnetic Detection

An electromagnetic offset module (EMOM) FMMD was used to measure the nonlinear magnetic moment of the synthesized particles at mixing frequencies. The details of the setup are mentioned in [35]. The settings of the excitation fields are summarized in Table 1.

Core size determination through the FMMD method was performed according to [38]. In this strategy, offset-dependent FMMD signals were utilized. Under the common assumption of a lognormal distribution, the measured signals were fitted by a nonlinear least square Levenberg–Marquardt described as follows:

(1) $$f_{L}\left(d_{c},d_{0},\sigma \right)=\frac{1}{\sqrt{2\pi }\cdot d_{c}\cdot \sigma }\cdot \exp\left(-\frac{ln^{2}\left(d_{c}/d_{0}\right)}{2\sigma^{2}}\right)$$

For every median of the distribution (*d*_0_), the best-fitting combination of the width of the distribution (*σ*) and the number of particles (*N_p_*) was determined. The parameters were then utilized to calculate the respective mass of iron (*m_Fe_*) for each distribution with

(2) $$m_{Fe}=\frac{3M_{Fe}}{3M_{Fe}+4M_{O}}\cdot \rho_{Fe_{3}O_{4}}\cdot N_{p}\cdot \frac{\pi }{6}\cdot \;d_{0}^{3}\;\exp\left(\frac{9\sigma^{2}}{2}\right)$$

using the molar mass of iron *M*_Fe_ = 55.845 g/mol and oxygen, *M*_0_ = 15.99 g/mol.

Finally, the experimental determination of the iron mass within the sample using ICP-OES allowed for the selection of the appropriate distribution.

## 3. Results

### 3.1. Synthesis and Characterization of Magnetic Nanoparticles

**Figure 2:**
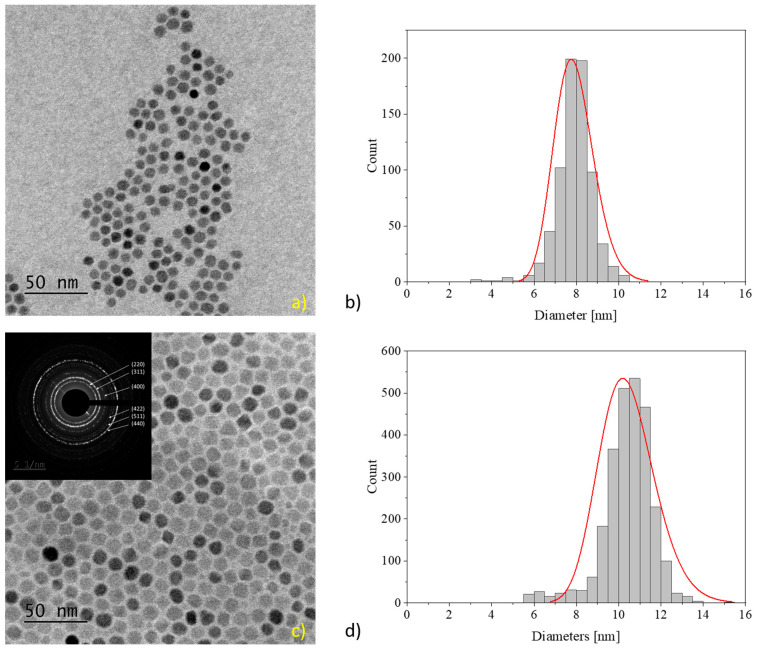
Sample of TEM images for the seed IONPs (**a**) NJ15 and (**c**) NJ19. The histogram and the distribution for respective particles are shown in (**b**) for NJ15 and (**d**) for NJ19.

IONPs synthesized by the thermal decomposition method generally represent a highly crystalline core of Fe_3_O_4_, which includes a narrow amorphous surface boundary consisting of Fe_x_O_y_ and a coating of oleic acid and oleylamine molecules, which renders the particles hydrophobic. Due to the fact that these IONPs start to nucleate within a very short time interval, the as-synthesized IONPs show a narrow size distribution. The obtained seed IONPs showed two peaks in the DLS, one at the desired size of 10 nm and another peak at 250 nm, showing the presence of large precipitates (see Supplementary Materials). To remove these aggregates and reduce the size distribution, a size fractionation by the precipitation of the IONPs from hexane solution using ethanol was performed. After the precipitation and removal of the aggregates, the sample (called NJ15) showed a hydrodynamic radius of 12.1 nm with a PDI of 5.9%. The TEM images showed spherical IONPs arranged in densely packed two-dimensional arrays (Figure 2a). The average size of the IONPs determined by ImageJ [44] was 7.9 ± 0.7 nm (Figure 2b). These seed particles were grown and purified, and the resultant sample NJ19 was analyzed by DLS and TEM (Figure 2c). The hydrodynamic radius increased up to 21.6 nm, and the size determined by size evaluation of the TEM images was 10.6 ± 1.1 nm (Figure 2d), see Table 2. Selected area electron diffraction allowed the inverse spinel structure to be indexed, indicating that the received particles consist of magnetite (inset of Figure 2c). Under the assumption that all the Fe(acac)_3_ in the reaction mixture were transformed quantitatively into Fe_3_O_4_ and added to the seed crystals, the diameter *d_IONP_* of the grown particles could be estimated by the formula given in Equation (3) [45].

(3) $$d_{IONP}^{3}=d_{seed}^{3}+\frac{6\cdot 10^{21}\cdot m_{Fe}}{\pi \cdot \rho_{Fe_{3}O_{4}}\cdot n_{seed}}$$

Here, *m_Fe_* is the mass of Fe in Fe(acac)_3_, ρ_Fe3O4_ denotes the density of Fe_3_O_4_ (5.2 g/cm^3^), and *n_seed_* is the number of seed particles employed in the reaction.

With a diameter *d_seed_* of 7.9 nm, *m_Fe_* of 112 mg, and *n_seed_* of 4.1 × 10^16^, we calculated a new diameter *d_IONP_* of 11.4 nm, which is in good accordance with the experimentally derived result.

### 3.2. Determination of the IONP’s Size by FMMD

The samples NJ15 and NJ19 were prepared by diluting 40 µL of the stock solution into 100 µL of toluene. The nonlinear magnetic moment response signal in FMMD originates from the underlying relaxation mechanisms, i.e., Néel and Brownian relaxations. Hence, when aiming for core size analysis, the samples were initially dried out to suppress the Brownian relaxation contribution, yielding a response signal that was mainly Néel relaxation-dominated, which refers to the rotation of the magnetic moment in the crystalline core.

**Figure 3:**
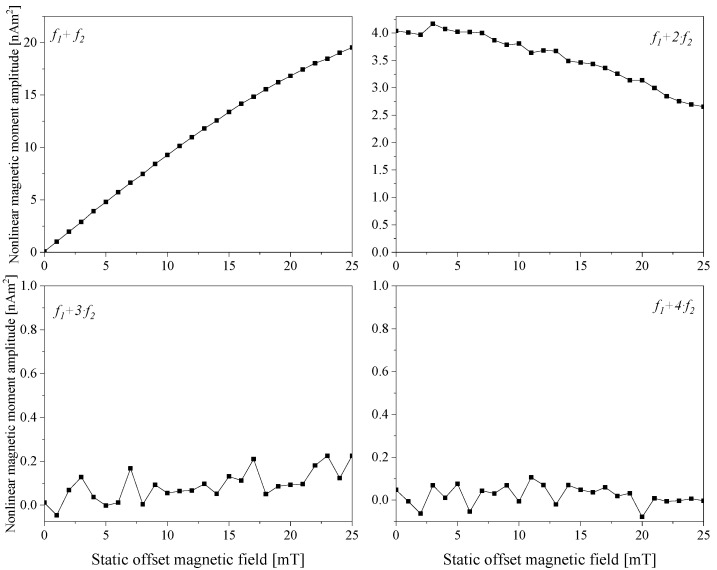
Measured nonlinear magnetic moment responses of the sample NJ15 in an immobilized state at mixing frequencies, *f*_1_ *+ f*_2_ (**top left**), *f*_1_ *+* 2⋅*f*_2_ (**top right**), *f*_1_ *+* 3⋅*f*_2_ (**bottom left**)_,_ and *f*_1_ + 4⋅*f*_2_ (**bottom right**), over the static magnetic offset field range from 0 mT to 25 mT.

The samples were measured using the EMOM setup introduced in Section 2.3. Figure 3 depicts the nonlinear magnetic moment amplitude response of the first four mixing harmonics (*f*_1_ *+ f*_2_, *f*_1_ *+* 2⋅*f*_2_, *f*_1_ *+* 3⋅*f*_2_ and *f*_1_ *+* 4⋅*f*_2_) for the sample NJ15. Here, the magnetic response signal was mainly observed at the first two harmonics, and the measurement signals at *f*_1_ + 3·*f*_2_ and *f*_1_ + 4·*f*_2_ fluctuated around the zero level. Moreover, the characteristic features of the signals (i.e., minima, maxima, and zero crossings) were not seen since they were out of the scanning range of the static offset magnetic field.

**Figure 4:**
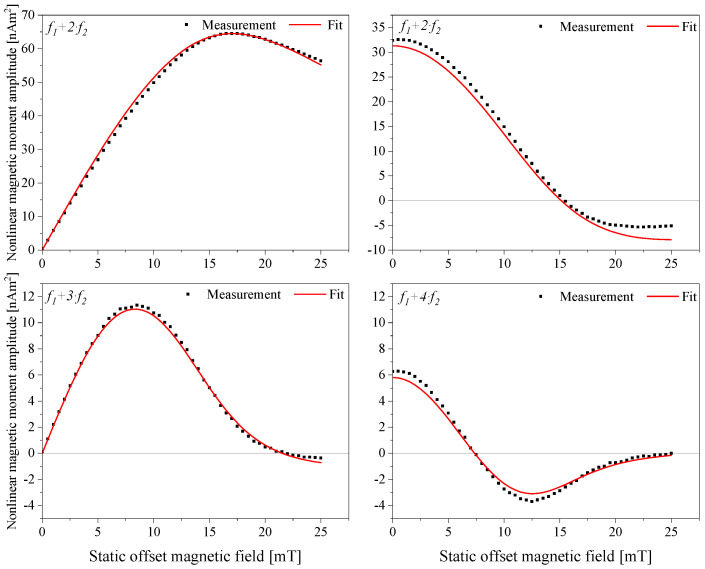
Measured nonlinear magnetic moment responses of the sample NJ19 in an immobilized state at mixing frequencies, *f*_1_ *+ f*_2_ (**top left**), *f*_1_ *+* 2⋅*f*_2_ (**top right**), *f*_1_ *+* 3⋅*f*_2_ (**bottom left**)_,_ and *f*_1_ + 4⋅*f*_2_ (**bottom right**), over a static magnetic offset field range from 0 mT to 25 mT. The black-filled squares represent the measurement data of the sample, and the solid red line represents the fitting to the respective measurement data.

**Figure 5:**
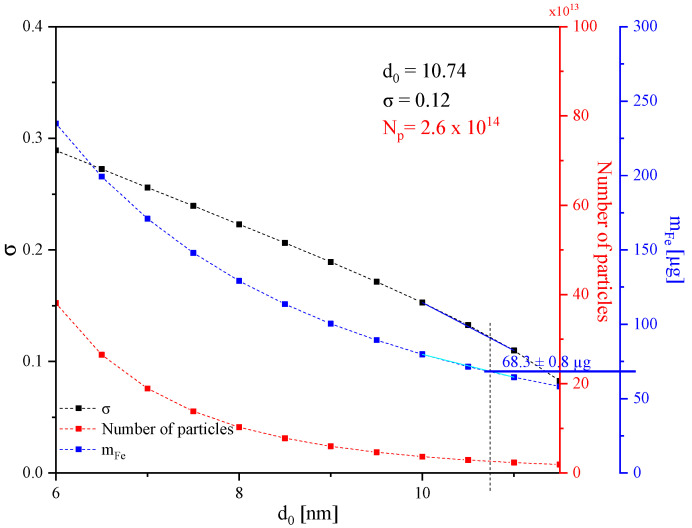
Look-up graph of all the two-parameter fits performed for various d_0_′s compared to the measured data of sample NJ19 leading to R^2^ > 0.99. The σ values are depicted as black squares. The plot features σ values as black squares, particle count (N_P_) as red squares, and calculated iron mass (*m_Fe_*) as blue squares. The iron mass obtained via ICP-OES is indicated by a blue line. To enhance visual clarity, lines connect corresponding points for each parameter set.

The measurement results of the NJ19 sample are presented in Figure 4. An adequate magnetic response is observed across all four harmonics, and the characteristic features of the FMMD signals are clearly visible. This allows us to proceed to the next step of the analysis, the core size analysis, by fitting the measurement signal and correlating it to the iron content present in the sample. The fitting of the measured signal with the assumption of lognormally distributed core sizes and respective magnetic moments was performed using an in-house LabVIEW program, as described in [26]. The lookup graph of the best-fit parameters (σ and N_p_) for different d_0_ values yielding an R^2^ > 0.99 is depicted in Figure 5. Here, the distribution width σ, the number of particles (N_p_), and the calculated iron mass values were plotted, respectively, in black, red, and blue solid squares. The iron mass (m_Fe_) was calculated for every distribution according to Equation (2) and plotted for the respective distributions. The coinciding point with the iron mass experimentally determined by ICP-OES is denoted in the graph as a solid blue line, intersecting the y-axis associated with the iron mass.

The experimentally determined iron mass allows for the selection of the best-fitting distribution. In the case of NJ19, the analysis revealed that *d*_0_ = 10.74 nm, σ = 0.12, and *N_p_* = 2.6·10^14^. A comparative assessment of the FMMD results with TEM results corroborates our findings, showcasing an excellent agreement in both the core diameter and the width of the particle size distribution.

The experimental results clearly demonstrate that an increase in core sizes leads to a significant enhancement of the signal. Specifically, the comparison of the maxima of the *f*_1_ *+* 2·*f*_2_ signal indicates an approximately 8-fold increase in signal strength. Additionally, there was a noticeable improvement in the signal response from the larger batch of particles. Based on these findings, it is suggested that for monocore iron oxide particles to provide reliable signals in bio-sensing applications, a lower size limit of at least 10 nm should be maintained. Reducing the core size below this threshold may result in suboptimal signal performance.

## 4. Conclusions

We reported on the response of two differently sized IONP samples in FMMD measurements. We found that the IONP sample with a diameter of 7.8 ± 0.7 nm (NJ15) exhibited a significantly diminished magnetic response. On the contrary, the sample with a size of 10.2 ± 1.1 nm (NJ19), characterized by a larger diameter, generated a magnetic signal sufficient for the FMMD analysis. Notably, the disparity in diameters between these two samples was a mere 2.4 nm. This understanding underscores the sensitivity of the magnetic response to particle size variation within IONPs, affirming the critical role of precise size characterization in magnetic nanoparticle research. The alignment of our findings by FMMD with TEM analyses validates our experimental approach. Additionally, the FMMD can be incorporated into the synthesis procedures for the evaluation of MNP production in different stages of synthesis. Looking forward, we propose that this technique be used for the inline monitoring of MNP production due to its ease of integration.

## Figures and Tables

**Table 1:** Settings of the frequency mixing magnetic detection setup.

	**Frequency *f*_i_ [Hz]**	**Magnetic Field Amplitude *B*_i_ [mT]**
**Low-frequency field (*B*_2_)**	60	16
**High-frequency field (*B*_1_)**	40,500	1.2
**Static offset field (*B*_0_)**	-	0, 1, … 24 (25 steps)

**Table 2:** Summary of characterization data of differently sized IONPs.

Sample	Hydrodynamic Size from DLS [nm]	Size from TEM [nm]	StDev from TEM [nm]
NJ15	12.1	7.8	0.7
NJ19	21.6	10.2	1.1

## Data Availability

The data presented in this study are available on request from the corresponding author.

## Supplementary Materials

The following supporting information can be downloaded at: https://www.mdpi.com/article/10.3390/s24134223/s1, Figure S1: Plot showing the intensity weighted DLS spectra for the samples NH15 and NH19.

## References

[1] Baki A., Wiekhorst F., Bleul R. (2021). Advances in Magnetic Nanoparticles Engineering for Biomedical Applications—A Review. Bioengineering.

[2] Ali A., Shah T., Ullah R., Zhou P., Guo M., Ovais M., Tan Z., Rui Y. (2021). Review on Recent Progress in Magnetic Nanoparticles: Synthesis, Characterization, and Diverse Applications. Front. Chem..

[3] Avasthi A., Caro C., Pozo-Torres E., Leal M.P., García-Martín M.L. (2020). Magnetic Nanoparticles as MRI Contrast Agents. Top. Curr. Chem..

[4] Dadfar S.M., Camozzi D., Darguzyte M., Roemhild K., Varvarà P., Metselaar J., Banala S., Straub M., Güvener N., Engelmann U. (2020). Size-Isolation of Superparamagnetic Iron Oxide Nanoparticles Improves MRI, MPI and Hyperthermia Performance. J. Nanobiotechnol..

[5] Buzug T.M., Borgert J. (2012). Magnetic Particle Imaging: A Novel SPIO Nanoparticle Imaging Technique.

[6] Gehrke N., Heinke D., Eberbeck D., Ludwig F., Wawrzik T., Kuhlmann C., Briel A. (2015). Magnetic Characterization of Clustered Core Magnetic Nanoparticles for MPI. IEEE Trans. Magn..

[7] Herynek V., Babič M., Kaman O., Charvátová H., Veselá M., Šefc L. (2020). Development of Novel Nanoparticles for MPI. Int. J. Magn. Part. Imaging.

[8] Engelmann U.M., Fitter J.L., Baumann M. (2019). Assessing Magnetic Fluid Hyperthermia: Magnetic Relaxation Simulation, Modeling of Nanoparticle Uptake inside Pancreatic Tumor Cells and in Vitro Efficacy.

[9] Das P., Colombo M., Prosperi D. (2019). Recent Advances in Magnetic Fluid Hyperthermia for Cancer Therapy. Colloids Surf. B Biointerfaces.

[10] Hedayatnasab Z., Abnisa F., Daud W.M.A.W. (2017). Review on Magnetic Nanoparticles for Magnetic Nanofluid Hyperthermia Application. Mater. Des..

[11] Beković M., Ban I., Drofenik M., Stergar J. (2023). Magnetic Nanoparticles as Mediators for Magnetic Hyperthermia Therapy Applications: A Status Review. Appl. Sci..

[12] Chen Y.-T., Kolhatkar A.G., Zenasni O., Xu S., Lee T.R. (2017). Biosensing Using Magnetic Particle Detection Techniques. Sensors.

[13] Wang Z., Wei L., Chen Y. (2024). Magnetic Particles-Integrated CRISPR/Cas Systems for Biosensing. TrAC Trends Anal. Chem..

[14] Wei L., Wang Z., Zhang H., Jiang F., Chen Y. (2024). Recent Advances in Magnetic Relaxation Switching Biosensors for Animal-Derived Food Safety Detection. Trends Food Sci. Technol..

[15] Tegafaw T., Liu S., Ahmad M.Y., Saidi A.K.A.A., Zhao D., Liu Y., Nam S.-W., Chang Y., Lee G.H. (2023). Magnetic Nanoparticle-Based High-Performance Positive and Negative Magnetic Resonance Imaging Contrast Agents. Pharmaceutics.

[16] Zhang Q., Yin R., Guan G., Liu H., Song G. (2024). Renal Clearable Magnetic Nanoparticles for Magnetic Resonance Imaging and Guided Therapy. WIREs Nanomed. Nanobiotechnology.

[17] Rodriguez B., Rivera D., Zhang J.Y., Brown C., Young T., Williams T., Huq S., Mattioli M., Bouras A., Hadjpanayis C.G. (2024). Magnetic Hyperthermia Therapy for High-Grade Glioma: A State-of-the-Art Review. Pharmaceuticals.

[18] Machala L., Zboril R., Gedanken A. (2007). Amorphous Iron(III) OxideA Review. J. Phys. Chem. B.

[19] Berkowitz A.E., Schuele W.J., Flanders P.J. (1968). Influence of Crystallite Size on the Magnetic Properties of Acicular γ-Fe_2_O_3_ Particles. J. Appl. Phys..

[20] Selim M.M., El-Safty S., Tounsi A., Shenashen M. (2024). A Review of Magnetic Nanoparticles Used in Nanomedicine. APL Mater..

[21] Wu K., Su D., Saha R., Liu J., Chugh V.K., Wang J.-P. (2020). Magnetic Particle Spectroscopy: A Short Review of Applications Using Magnetic Nanoparticles. ACS Appl. Nano Mater..

[22] Marć M., Wolak W., Drzewiński A., Mudry S., Shtablavyi I., Dudek M.R. (2024). The Concept of Using 2D Self-Assembly of Magnetic Nanoparticles for Bioassays. Appl. Sci..

[23] Wu K., Chugh V.K., Krishna V.D., Wang Y.A., Gordon T.D., Cheeran M.C.-J., Wang J.-P. (2022). Five-Minute Magnetic Nanoparticle Spectroscopy-Based Bioassay for Ultrafast Detection of SARS-CoV-2 Spike Protein. ACS Appl. Nano Mater..

[24] Orlov A.V., Bragina V.A., Nikitin M.P., Nikitin P.I. (2016). Rapid Dry-Reagent Immunomagnetic Biosensing Platform Based on Volumetric Detection of Nanoparticles on 3D Structures. Biosens. Bioelectron..

[25] Smolensky E.D., Park H.-Y.E., Zhou Y., Rolla G.A., Marjańska M., Botta M., Pierre V.C. (2013). Scaling Laws at the Nanosize: The Effect of Particle Size and Shape on the Magnetism and Relaxivity of Iron Oxide Nanoparticle Contrast Agents. J. Mater. Chem. B.

[26] Engelmann U.M., Pourshahidi A.M., Shalaby A., Krause H.J. (2022). Probing Particle Size Dependency of Frequency Mixing Magnetic Detection with Dynamic Relaxation Simulation. J. Magn. Magn. Mater..

[27] Wu K., Liu J., Saha R., Peng C., Su D., Wang Y.A., Wang J.-P. (2021). Investigation of Commercial Iron Oxide Nanoparticles: Structural and Magnetic Property Characterization. ACS Omega.

[28] Wu K., Liu J., Chugh V.K., Liang S., Saha R., Krishna V.D., Cheeran M.C.-J., Wang J.-P. (2022). Magnetic Nanoparticles and Magnetic Particle Spectroscopy-Based Bioassays: A 15 Year Recap. Nano Futures.

[29] Krause H.-J., Wolters N., Zhang Y., Offenhäusser A., Miethe P., Meyer M.H.F., Hartmann M., Keusgen M. (2007). Magnetic Particle Detection by Frequency Mixing for Immunoassay Applications. J. Magn. Magn. Mater..

[30] Wu K., Chugh V.K., di Girolamo A., Liu J., Saha R., Su D., Krishna D., Nair A., Davies W., Wang A.Y. (2021). Portable Magnetic Particle Spectrometer (MPS) for Future Rapid and Wash-Free Bioassays. ACS Appl. Mater. Interfaces.

[31] Abuawad A., Ashhab Y., Offenhäusser A., Krause H.-J. (2023). DNA Sensor for the Detection of Brucella Spp. Based on Magnetic Nanoparticle Markers. Int. J. Mol. Sci..

[32] Hong H.-B. (2011). Detection of Two Different Influenza A Viruses Using a Nitrocellulose Membrane and a Magnetic Biosensor. J. Immunol. Methods.

[33] Wu K., Liu J., Saha R., Su D., Krishna V.D., Cheeran M.C.-J., Wang J.-P. (2020). Magnetic Particle Spectroscopy for Detection of Influenza A Virus Subtype H1N1. ACS Appl. Mater. Interfaces.

[34] Achtsnicht S., Pourshahidi A.M., Offenhäusser A., Krause H.-J. (2019). Multiplex Detection of Different Magnetic Beads Using Frequency Scanning in Magnetic Frequency Mixing Technique. Sensors.

[35] Pourshahidi A.M., Achtsnicht S., Nambipareechee M.M., Offenhäusser A., Krause H.-J. (2021). Multiplex Detection of Magnetic Beads Using Offset Field Dependent Frequency Mixing Magnetic Detection. Sensors.

[36] Wu K., Wang Y., Feng Y., Yu L., Wang J.-P. (2015). Colorize Magnetic Nanoparticles Using a Search Coil Based Testing Method. J. Magn. Magn. Mater..

[37] Chugh V.K., Liang S., Yari P., Wu K., Wang J.-P. (2023). A Method for Multiplexed and Volumetric-Based Magnetic Particle Spectroscopy Bioassay: Mathematical Study. J. Phys. D Appl. Phys..

[38] Pourshahidi A.M., Engelmann U.M., Offenhäusser A., Krause H.J. (2022). Resolving Ambiguities in Core Size Determination of Magnetic Nanoparticles from Magnetic Frequency Mixing Data. J. Magn. Magn. Mater..

[39] Pourshahidi A.M., Achtsnicht S., Offenhäusser A., Krause H.-J. (2022). Frequency Mixing Magnetic Detection Setup Employing Permanent Ring Magnets as a Static Offset Field Source. Sensors.

[40] Engelmann U.M., Simsek B., Shalaby A., Krause H.-J. (2024). Key Contributors to Signal Generation in Frequency Mixing Magnetic Detection (FMMD): An In Silico Study. Sensors.

[41] Massart R. (1981). Preparation of Aqueous Magnetic Liquids in Alkaline and Acidic Media. IEEE Trans. Magn..

[42] Sun S., Zeng H. (2002). Size-Controlled Synthesis of Magnetite Nanoparticles. J. Am. Chem. Soc..

[43] Sun S., Zeng H., Robinson D.B., Raoux S., Rice P.M., Wang S.X., Li G. (2004). Monodisperse MFe_2_ O_4_ (M = Fe, Co, Mn) Nanoparticles. J. Am. Chem. Soc..

[44] Schneider C.A., Rasband W.S., Eliceiri K.W. (2012). NIH Image to ImageJ: 25 Years of Image Analysis | Nature Methods. Nat. Methods.

[45] Leng W., Pati P., Vikesland J.P. (2015). Room Temperature Seed Mediated Growth of Gold Nanoparticles: Mechanistic Investigations and Life Cycle Assesment. Environ. Sci. Nano.

