# Supplementary material for: Impact of Particle Size on the Nonlinear Magnetic Response of Iron Oxide Nanoparticles during Frequency Mixing Magnetic Detection
Source: Sensors (Basel). 2024 Jun 29;24(13):4223. doi: 10.3390/s24134223 (PMC11244231; doi:10.3390/s24134223)

Supplementary Information to

Impact of Particle Size on the Nonlinear Magnetic Response of Iron Oxide Nanoparticles in Frequency mixing magnetic detection

Ali Mohammad Pourshahidi [1*], Neha Jean [1], Corinna Kaulen [2], Simon Jakobi [3], and Hans-Joachim Krause [1*]

[1] Institute of Biological Information Processing, Bioelectronics (IBI-3), Forschungszentrum Jülich, Germany; n.jean@fz-juelich.de (N.J.)

[2] Ostbayerische Technische Hochschule Regensburg,Germany; corinna.kaulen@oth-regensburg.de (C.K.)

[3] RWTH Aachen, Institute of Inorganic Chemistry,Germany; simon.jakobi@rwth-aachen.de (S.J.)

* Correspondence: a.pourshahidi@fz-juelich.de (A.M.P.); h.-j.krause@fz-juelich.de (H.-J.K.); Tel.:+49-2461-2955

Plot showing the intensity weighted DLS spectra for the samples NH15 and NH19:

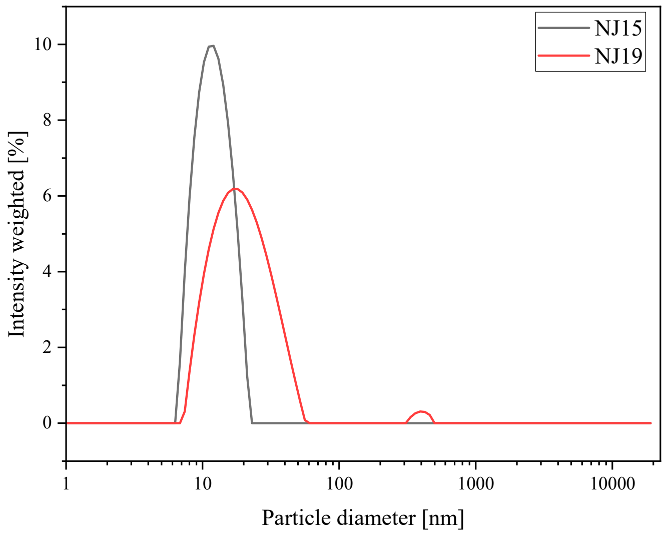

Supplement: Supplementary file 1 [file sensors-24-04223-s001.zip › sensors-3004637-supplementary.pdf]
